# Subcutaneous *Mycobacterium avium* complex infection in an adolescent with mixed connective tissue disease receiving mycophenolate mofetil

**DOI:** 10.1186/1546-0096-10-S1-A99

**Published:** 2012-07-13

**Authors:** Josephine Isgro, Patricia Costa Reis, Amy J Starr, Lisa F Imundo, Andrew H Eichenfield

**Affiliations:** 1 Morgan Stanley Children's Hospital of New York Presbyterian Hospital, Columbia University Medical Center, New York, NY, USA; 2 Santa Maria Hospital, Lisbon, Portugal

## Purpose

*Mycobacterium avium* complex (MAC) are non-tuberculous mycobacteria that are ubiquitously found in nature and can cause progressive pulmonary disease, superficial lymphadenitis, and disseminated infection in immunocompromised individuals. In this report, we present a case of disseminated MAC cutaneous disease in a patient with mixed connective tissue disease (MCTD) on immunosuppressive therapy.

## Results

### Case report

A 19 year-old adolescent male was diagnosed with mixed connective tissue disease in 2006 after presenting with progressive muscle weakness secondary to myositis, weight loss, diffuse lymphadenopathy, and anemia. Initial treatment included hydroxychloroquine and prednisone, to which mycophenolate mofetil (MMF) was added in 2009 with improved disease control. Approximately 9 months after starting MMF, the patient developed a number of soft tissue masses along the neck, anterior chest and abdominal walls, and upper extremities. Computerized tomography of the chest demonstrated multiple enlarged lymph nodes and subcutaneous nodules. Excisional biopsy of a supraclavicular nodule revealed focally necrotic fibroadipose tissue with extensive granulomatous inflammation and positive culture for *Mycobacterium avium* complex. MMF was discontinued and treatment with clarithromycin, ethambutol and rifabutin was started with good response.

## Conclusion

To our knowledge, this is the first reported case of disseminated soft tissue infection with MAC in a patient with connective tissue disease receiving mycophenolate mofetil.

## Disclosure

Josephine Isgro: None; Patricia Costa Reis: None; Amy J. Starr: None; Lisa F. Imundo: None; Andrew H. Eichenfield: None.

